# Anti-Inflammatory Effects of Vitamin D on Human Immune Cells in the Context of Bacterial Infection

**DOI:** 10.3390/nu8120806

**Published:** 2016-12-12

**Authors:** Edwin Hoe, Jordan Nathanielsz, Zheng Quan Toh, Leena Spry, Rachel Marimla, Anne Balloch, Kim Mulholland, Paul V. Licciardi

**Affiliations:** 1 Pneumococcal Research, Murdoch Childrens Research Institute, Melbourne VIC 3052, Australia; edwin.hoe@mcri.edu.au (E.H.); jordan.nathanielsz@mcri.edu.au (J.N.); zheng.quantoh@mcri.edu.au (Z.Q.T.); leena.spry@mcri.edu.au (L.S.); rachel.marimla@mcri.edu.au (R.M.); anne.balloch@mcri.edu.au (A.B.); kim.mulholland@lshtm.ac.uk (K.M.); 2 London School of Hygiene and Tropical Medicine, London WC1E 7HT, UK; 3 Department of Paediatrics, The University of Melbourne, Parkville VIC 3010, Australia

**Keywords:** vitamin D, inflammation, bacterial infection, pneumococcal

## Abstract

Vitamin D induces a diverse range of biological effects, including important functions in bone health, calcium homeostasis and, more recently, on immune function. The role of vitamin D during infection is of particular interest given data from epidemiological studies suggesting that vitamin D deficiency is associated with an increased risk of infection. Vitamin D has diverse immunomodulatory functions, although its role during bacterial infection remains unclear. In this study, we examined the effects of 1,25(OH)_2_D_3_, the active metabolite of vitamin D, on peripheral blood mononuclear cells (PBMCs) and purified immune cell subsets isolated from healthy adults following stimulation with the bacterial ligands heat-killed pneumococcal serotype 19F (HK19F) and lipopolysaccharide (LPS). We found that 1,25(OH)_2_D_3_ significantly reduced pro-inflammatory cytokines TNF-α, IFN-γ, and IL-1β as well as the chemokine IL-8 for both ligands (three- to 53-fold), while anti-inflammatory IL-10 was increased (two-fold, *p* = 0.016) in HK19F-stimulated monocytes. Levels of HK19F-specific IFN-γ were significantly higher (11.7-fold, *p* = 0.038) in vitamin D-insufficient adults (<50 nmol/L) compared to sufficient adults (>50 nmol/L). Vitamin D also shifted the pro-inflammatory/anti-inflammatory balance towards an anti-inflammatory phenotype and increased the CD14 expression on monocytes (*p* = 0.008) in response to LPS but not HK19F stimulation. These results suggest that 1,25(OH)_2_D_3_ may be an important regulator of the inflammatory response and supports further in vivo and clinical studies to confirm the potential benefits of vitamin D in this context.

## 1. Introduction

Global burden of disease data estimates that infections are responsible for ~3 million deaths of children under five years of age per year [1]. Bacterial infections comprise a significant proportion of this, with Gram-positive and Gram-negative bacteria such as *Streptococcus pneumoniae, Escherichia coli*, and *Salmonella typhi* the leading candidates [2]. Exposure to these organisms can be extremely high, especially in low-income country settings, leading to persistent activation of host inflammatory responses. Local immune responses in the early stages of infection, such as the recruitment of neutrophils and release of pro-inflammatory cytokines (IL-1β, TNF-α) play an important role in pathogen clearance. However, failure to clear the pathogen may lead to persistent infections and excessive inflammation, leading to serious diseases, such as pneumonia, meningitis, and diarrheal illness [2,3].

An increasing number of epidemiological studies have demonstrated a strong association between low levels of hydroxyvitamin D_3_ [25(OH)D3], the inactive precursor of vitamin D, and an increased risk of respiratory infections [4,5], especially in countries with low exposure to sunlight [6,7,8], while the data is less clear for enteric infections. In Mongolia, the high burden of pneumonia has been linked to vitamin D deficiency, and supplementation in young children protected against acute respiratory infection [9]. However, a recent review of randomized controlled trials of vitamin D supplementation for the prevention of respiratory infections was inconclusive [10].

Vitamin D has diverse immunomodulatory functions. The vitamin D receptor (VDR) is widely expressed on all immune cellular subsets and VDR ligation by vitamin D results in activation of key innate immune cells such as monocytes, macrophages, and neutrophils leading to enhanced chemotactic, phagocytic, and bactericidal activities [11,12,13]. The VDR binds 1,25(OH)_2_D_3_ with a much greater affinity than 25(OH)D_3_ [14]. Vitamin D also modulates the expression of Toll-like receptors (TLRs) [15] and the co-receptor CD14 [16] on important innate immune cells, promotes the conversion of 25(OH)D_3_ to the active form (dihydroxyvitamin D_3_—1,25(OH)_2_D_3_), and induces production of anti-microbial peptides, such as cathelicidin, which inhibit the growth of both Gram-positive and Gram-negative bacteria [17,18]. Vitamin D also promotes anti-inflammatory effects through up-regulation of IL-10 [19]. Therefore, it is likely that vitamin D has a critical role in modulating bacterial-specific inflammatory responses.

Given the paucity of data in relation to the immunomodulatory effects and possible protection elicited by vitamin D, we hypothesized that the active form of vitamin D, 1,25(OH)_2_D_3_, modulates critical host responses important in host protection against Gram-positive and Gram-negative infection.

## 2. Materials and Methods

### 2.1. Study Samples

Samples were collected from healthy adults (*n* = 19) aged 19–64 (mean = 30) years old from the Royal Children’s Hospital, Melbourne, and comprised six males and 13 females. The study was approved by the Royal Children’s Hospital Human Research Ethics Committee and all participants were recruited following informed consent.

### 2.2. Materials

The active metabolite of vitamin D_3_, 1,25(OH)_2_D_3_, and the inactive preform, 25(OH)D_3_, were purchased from Tocris Bioscience (Bristol, UK). Purified lipopolysaccharide (LPS) from *Escherichia coli* serotype 055:B5 was purchased from Sigma-Aldrich (St. Louis, MO, USA). Heat-killed pneumococcal bacteria serotype 19F (HK19F; reference strain originally obtained from the University of Alabama) was prepared by harvesting the mid-log phase of the bacterial culture and incubating in a water bath at 80 °C for 60 min. The bacterial concentration was determined before and after heat-killing by plating on horse blood agar plates. The HK19F was stored in aliquots at −80 °C prior to use, and the same batch was used throughout the study. Neutrophils were derived by stimulation of a HL-60 cell line (ATCC^®^ CCL-240™, Manassas, VA, USA) with 100 mM *N*,*N*-dimethylformamide for five days.

### 2.3. Cell Culture

Peripheral blood mononuclear cells (PBMCs) were isolated from heparinised whole blood using Lymphoprep (Axis-Shield, Oslo, Norway). CD14+ monocytes were isolated by negative selection using antibody conjugated magnetic beads (Stemcell Technologies, Tullamarine, VIC, Australia). The purity of the isolated CD14+ monocytes was 82%, consistent with manufacturer’s guidelines. PBMCs were cultured in duplicate using 24-well cell-culture plates (Thermo Scientific, Roskilde, Denmark). One million PBMCs/mL were pre-treated with either 100 nmol/L 1,25(OH)_2_D_3_, 500 nmol/L 25(OH)D_3_ or vehicle (RPMI-FBS medium containing 0.08% (*v*/*v*) ethanol; untreated) and incubated for 4 h at 37 °C in 5% CO_2_. Pre-treated PBMCs were then stimulated for 24 h with either LPS (1 μg/mL), HK19F (HK19F:PBMC ratio of 50:1) or unstimulated (RPMI-FBS). Similar experiments were performed for isolated CD14+ monocytes which were cultured in duplicate at 1.5 × 10^5^ cells/well in round-bottom 96-well culture plates (Greiner Bio-one, Frickenhausen, Germany). Supernatants were collected and stored at −80 °C until cytokine analysis.

### 2.4. Plasma 25(OH)D Measurement

Plasma samples from healthy volunteers were sent to the Peter MacCallum Cancer Institute, Melbourne, Australia to determine their 25(OH)D status. Plasma 25(OH)D concentrations were measured by a chemiluminescence delayed, one-step assay (Abbott Diagnostics, Wiesbaden, Germany) on the Abbott Architect ci4100 analyser. Three controls, Liquichek Speciality Control 1, 2, and 3 (Bio-Rad, Irvine, CA, USA) were included in all assays.

### 2.5. Measurement of Cytokines

Levels of TNF-α, IFN-γ, IL-1β, IL-10, IL-4, IL-8, and IL-17A in cultured supernatants from PBMCs and CD14+ monocytes were measured using a commercial Milliplex human cytokine multiplex bead array method, as per the manufacturer’s instructions (Milliplex; Millipore Corporation, Billerica, MA, USA).

### 2.6. Flow Cytometry

To identify specific immune cell subsets in PBMCs following 1,25(OH)_2_D_3_ treatment, the cells were stained with fluorescently-conjugated monoclonal antibodies CD4-BUV737, CD14-BV605, and CD3-BV510 (BD Bioscience; San Diego, CA, USA). Compensation bead particles were used to account for the spectral overlap (BD Bioscience, San Diego, CA, USA) above and analyzed using the BD LSRII flow cytometer. Unstained PBMCs were used as a control and a minimum of 20,000 events were analysed per sample gated on live, single cell lymphocyte gate based on FFS and SSC, where the expression of the cell surface molecules were evaluated using MFI values using BD FACSDiva 8.0.1 software (Becton, Dickinson and Company, Franklin Lakes, NJ, USA).

### 2.7. Neutrophil Migration Assay

Neutrophils cultured in a 24-well plate at a concentration of 4 × 10^6^ cells/mL. Cells were either pre-treated with 100 nmol/L of 1,25(OH)_2_D_3_ or left untreated and incubated at 37 °C, 5% CO_2_ for 1 h. Transwell plates (6.5 mm Transwell^®^ with a 8.0 μm pore polycarbonate membrane insert, Corning, NY, USA) were used to test neutrophil migration. The bottom chamber contained 400 μL of 5.2 mg/mL casein (as a chemoattractant) and the membrane was submerged in casein for 30 min at 37 °C and 5% CO_2_. Neutrophils (5 × 10^4^ cells) were added to the top chamber and incubated at 37 °C and 5% CO_2_ for 1 h. Following this, the top chambers were removed and the number of migrated cells were determined by cell counting using a haemocytometer.

### 2.8. Statistics

Cytokine data was presented as mean ± standard error of the mean (SEM). Flow cytometry and cytokine ratio data were presented as mean fold change ± SEM. Graphical presentation and statistical analysis were calculated using GraphPad Prism 6 software (Graphpad Software Inc., La Jolla, CA, USA). Comparison of cytokine data and ratios between treated groups (with 1,25(OH)_2_D_3_ or 25(OH)D_3_) and untreated groups were done using a non-parametric paired Wilcoxon sign-rank test. Flow cytometry data was analysed by Mann-Whitney U test. An unpaired Student’s *t*-test was used to compare cytokine levels between individuals with an insufficient (<50 nmol/L) and sufficient (>50 nmol/L) 25(OH)D status. Spearman’s correlation test was performed for plasma 25(OH)D and cytokine correlations. All tests were two-tailed with a *p*-value < 0.05 considered significant.

## 3. Results

### 3.1. 1,25(OH)_2_D_3_ Suppresses Pro-Inflammatory Cytokines in PBMCs and CD14+ Monocytes

**Figure 1:**
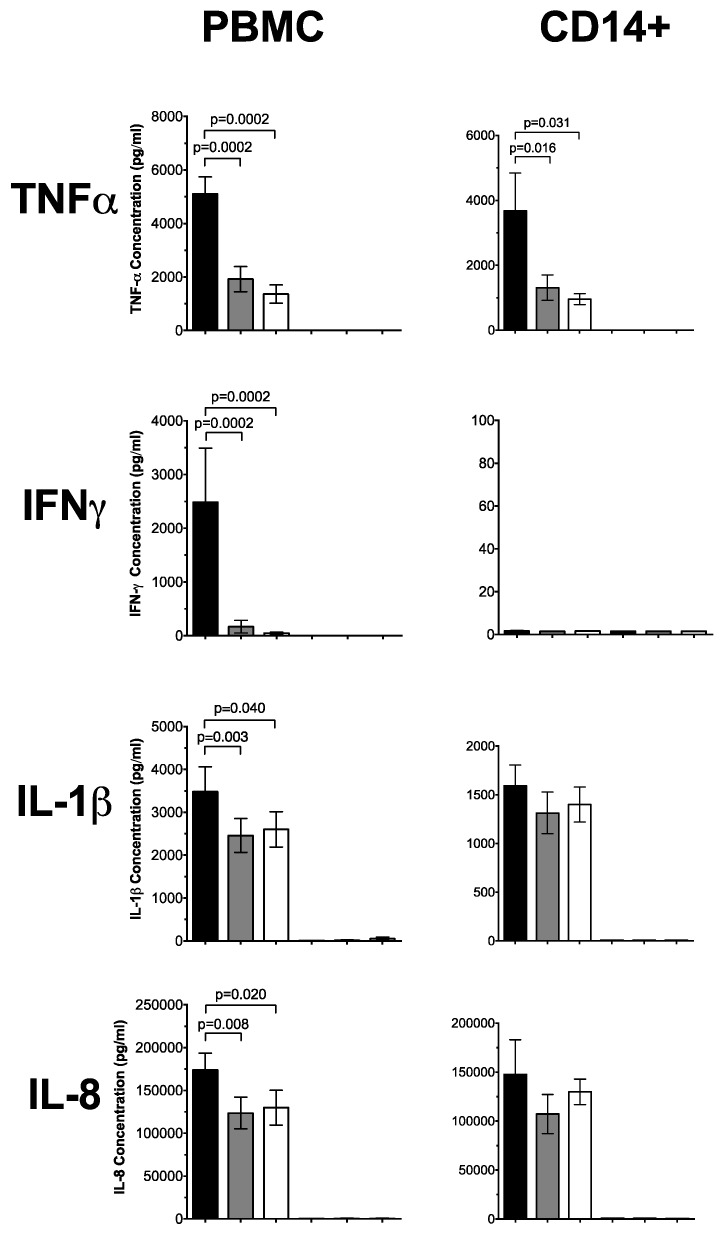

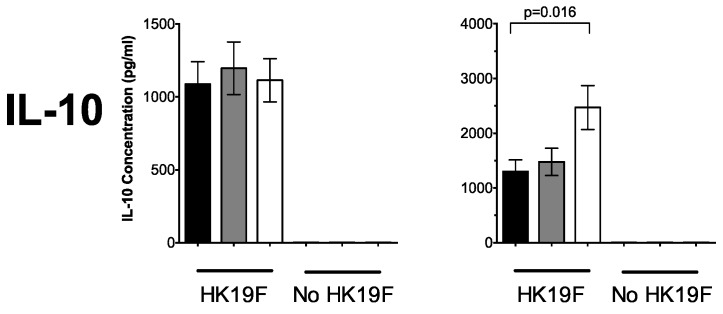
Cytokine responses of HK19F-stimulated PBMCs and CD14+ monocytes. HK19F (1:50 ratio) stimulated PBMCs and CD14+ monocytes pre-treated with 1,25(OH)_2_D_3_ or 25(OH)D_3_ for 4 h. Cytokine concentrations (pg/mL) were measured in PBMC and CD14+ monocyte supernatants by multiplex assay. Data shown represents Mean ± SEM for PBMCs (*n* = 13) and CD14+ monocytes (*n* = 7), respectively. Significance was calculated using a paired Wilcoxon sign-rank test (black bar = untreated, grey bar = 25(OH)D_3_ and white bar = 1,25(OH)_2_D_3_).

The cytokine response in PBMCs pre-treated with 1,25(OH)_2_D_3_ and stimulated with HK19F is shown in Figure 1, and shows a significant reduction in TNF-α (3.7-fold, *p* = 0.0002) and IFN-γ (53-fold, *p* = 0.0002) compared to untreated, HK19F-stimulated cells. IL-1β (*p* = 0.04) and IL-8 (*p* = 0.02) were also reduced by 1,25(OH)_2_D_3_ while IL-10 was unaffected by 1,25(OH)_2_D_3_ or 25(OH)D_3_. In HK19F-stimulated CD14+ monocytes 1,25(OH)_2_D_3_ significantly decreased TNF-α (four-fold, *p* = 0.031) but increased IL-10 (two-fold, *p* = 0.016) compared to untreated HK19F-stimulated CD14+ monocytes; no significant differences were observed for IFN-γ, IL-1β, or IL-8 levels. Negligible concentrations of IL-4 and IL-17A were detected in these supernatants.

**Figure 2:**
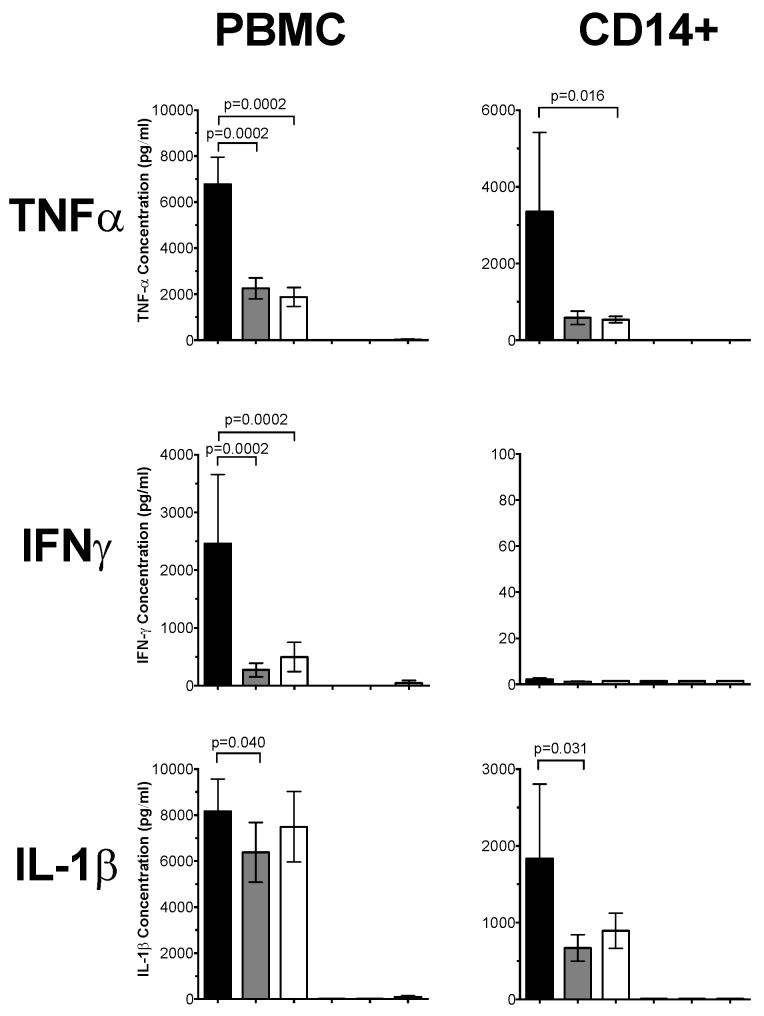

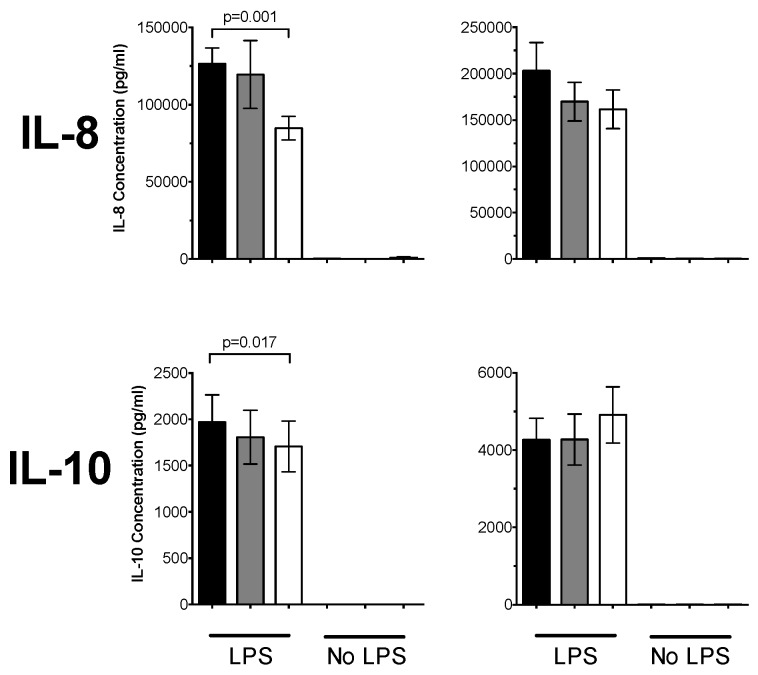
Cytokine responses of LPS-stimulated PBMCs and CD14+ monocytes. LPS (1 μg/mL) stimulated PBMCs and CD14+ monocytes pre-treated with 1,25(OH)_2_D_3_ or 25(OH)D_3_ for 4 h. Cytokine concentrations (pg/mL) were measured in PBMC and CD14+ monocyte supernatants by multiplex assay. Data shown represents the mean ± SEM for PBMCs (*n* = 13) and CD14+ monocytes (*n* = 7), respectively. Significance was calculated using a paired Wilcoxon sign-ranked test (black bar = untreated, grey bar = 25(OH)D_3_ and white bar = 1,25(OH)_2_D_3_).

For PBMCs stimulated with LPS, TNF-α was significantly reduced by 3.6-fold and three-fold by 1,25(OH)_2_D_3_ and 25(OH)D_3_, respectively (*p* = 0.0002; Figure 2). The IFN-γ concentrations were also significantly reduced in PBMCs treated with 25(OH)D_3_ (9-fold, *p* = 0.0002) and 1,25(OH)_2_D_3_ (five-fold, *p* = 0.0002) compared to untreated PBMCs. PBMCs treated with 25(OH)D_3_ significantly reduced IL-1β (1.3-fold; *p* = 0.04), whereas PBMCs treated with 1,25(OH)_2_D_3_ significantly reduced IL-10 (1.2-fold, *p* = 0.017) and IL-8 (1.5-fold; *p* = 0.001). In LPS-stimulated CD14+ monocytes, 1,25(OH)_2_D_3_ significantly reduced TNF-α by 6.2-fold (*p* = 0.016) compared to untreated monocytes. The CD14+ monocytes treated with 25(OH)D_3_ significantly reduced IL-1β by 2.7-fold (*p* = 0.031), but not for 1,25(OH)_2_D_3_.

There was no differential effect of 1,25(OH)_2_D_3_ or 25(OH)D_3_ when we compared the responses after stratification by gender or age (data not shown), although the numbers of individuals for each of these sub-analyses were low.

**Figure 3:**
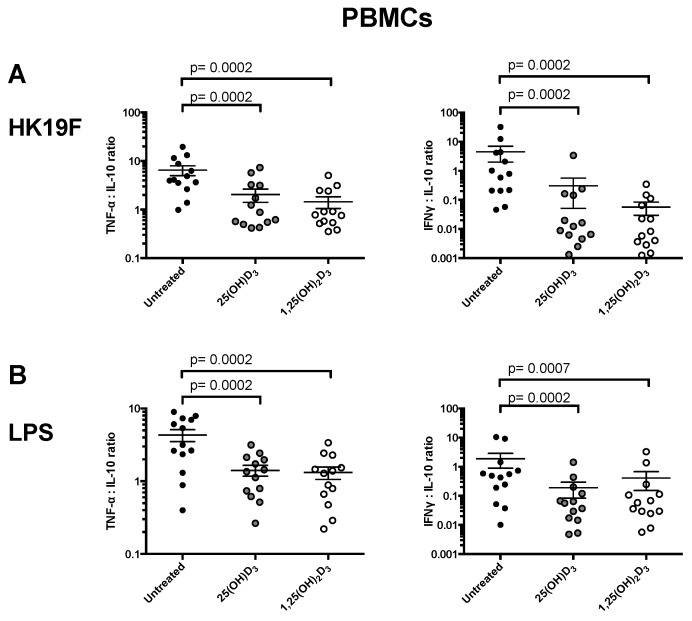

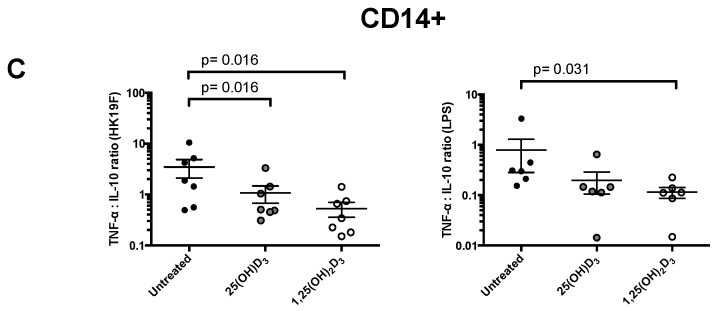
Effect of 1,25(OH)_2_D_3_ on PBMCs and CD14+ monocytes cytokine profile. PBMCs pre-treated with 1,25(OH)_2_D_3_ or 25(OH)D_3_ for 4 h and stimulated with (**A**) HK19F or (**B**) LPS. Data shown represents the mean ± SEM, (*n* = 13); and (**C**) CD14+ monocytes pre-treated with 1,25(OH)_2_D_3_ or 25(OH)D_3_ for 4 h and stimulated with HK19F or LPS. Data shown represents the mean ± SEM (*n* = 7). Treatment groups were compared with untreated-stimulated groups only, with significance calculated using a paired Wilcoxon sign-rank test.

**Figure 4:**
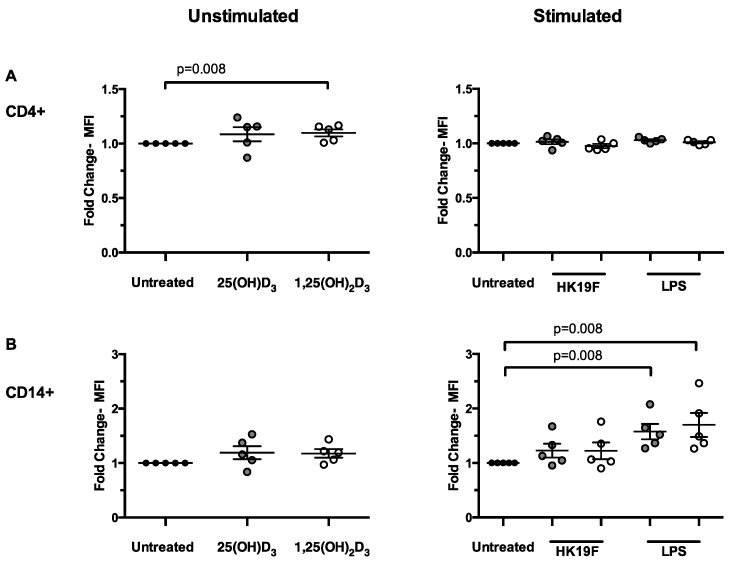
Effects of 1,25(OH)_2_D_3_ pre-treatment on the immunophenotype of PBMCs. Fold change in median fluorescence intensity (MFI) for (**A**) CD4 and (**B**) CD14 in PBMCs (*n* = 5) pre-treated with 1,25(OH)_2_D_3_ or 25(OH)D_3_ in comparison to unstimulated and HK19F/LPS stimulation. Data represents the mean fold change ± SEM. Significance was calculated using a Mann-Whitney test (black bar = untreated, grey bar = 25(OH)D_3_ and white bar = 1,25(OH)_2_D_3_).

We investigated whether 1,25(OH)_2_D_3_ or 25(OH)D_3_ modulates the pro-inflammatory/anti-inflammatory balance in response to TLR stimulation. In HK19F-stimulated PBMCs, 1,25(OH)_2_D_3_ shifted the cytokine ratio towards an anti-inflammatory phenotype by 4.5-fold (*p* = 0.0002) for TNF-α/IL-10 ratio, while for the IFN-γ/IL-10 ratio, this was decreased by 79-fold (*p* = 0.0002) (Figure 3). For LPS-stimulated PBMCs, a similar effect was observed with 1,25(OH)_2_D_3_ in comparison to untreated groups for TNF-α/IL-10 (*p* = 0.0002) and for IFN-γ/IL-10 (*p* = 0.0002) (Figure 4B). A shift towards an anti-inflammatory phenotype was also observed for TNF-α/IL-10 ratio in HK19F (6.5-fold, *p* = 0.016) and LPS (7.2-fold, *p* = 0.031) stimulated CD14+ monocytes treated with 1,25(OH)_2_D_3_ (Figure 3).

### 3.2. 1,25(OH)_2_D_3_ Enhances CD14 Expression in Stimulated PBMCs

We next examined the phenotype of key immune cell subsets in PBMCs following pre-treatment with 1,25(OH)_2_D_3_ or 25(OH)D_3_. In unstimulated PBMCs, 1,25(OH)_2_D_3_ significantly increased CD4 expression on T cells (*p* = 0.008; Figure 4A) while for HK19F, 1,25(OH)_2_D_3_ had no effect on CD4 or CD14 expression (Figure 4A,B). For LPS-stimulated PBMCs, expression of CD14 was increased 1.7-fold (*p* = 0.008) by treatment with 1,25(OH)_2_D_3_ and 25(OH)D_3_, but no change was observed for CD4 expression (Figure 4A).

### 3.3. Plasma 25(OH)D Status Influences the Magnitude of Inflammatory Response

**Figure 5:**
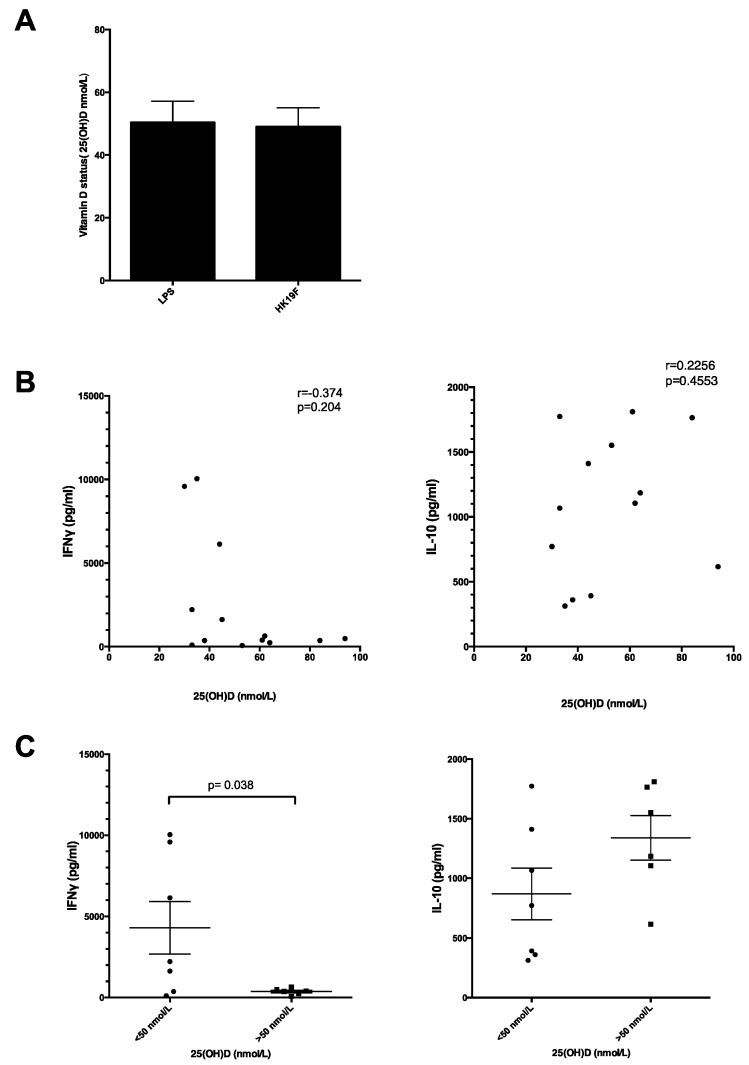
Influence of 25(OH)D status on inflammatory responses. (**A**) Data shows mean ± SEM serum level of 25(OH)D in nmol/L for individuals in the LPS (*n* = 13) and HK19F (*n* = 13) conditions; (**B**) correlation between plasma 25(OH)D status of healthy adults and levels of cytokines (IFN-γ and IL-10) (*n* = 13). Correlation was calculated using Spearman’s test; and (**C**) correlation between cytokine levels (IFN-γ and IL-10) and plasma 25(OH)D status of healthy adults with >50 nmol/L (*n* = 7) and <50 nmol/L (*n* = 6). Significance was calculated using an unpaired *t*-test.

We then examined whether circulating vitamin D status affected cytokine responses following HK19F and LPS stimulation. There was a similar 25(OH)D status between individuals used for each experiment (Figure 5A). Although no significant correlation was found between plasma 25(OH)D levels and IFN-γ concentrations (Figure 5B), individuals with 25(OH)D insufficiency (<50 nmol/L) exhibited 11.7-fold (*p* = 0.038) higher IFN-γ following HK19F stimulation compared to 25(OH)D sufficient (>50 nmol/L) individuals (Figure 5C). There was a weak correlation between plasma 25(OH)D levels and IL-10 concentrations (*r* = 0.2256; Figure 5B) with 25(OH)D sufficient individuals producing 1.5-fold higher IL-10 levels following HK19F stimulation compared to 25(OH)D insufficient individuals, although not significant (Figure 5C).

### 3.4. 1,25(OH)_2_D_3_ Regulates Neutrophil Migration

**Figure 6:**
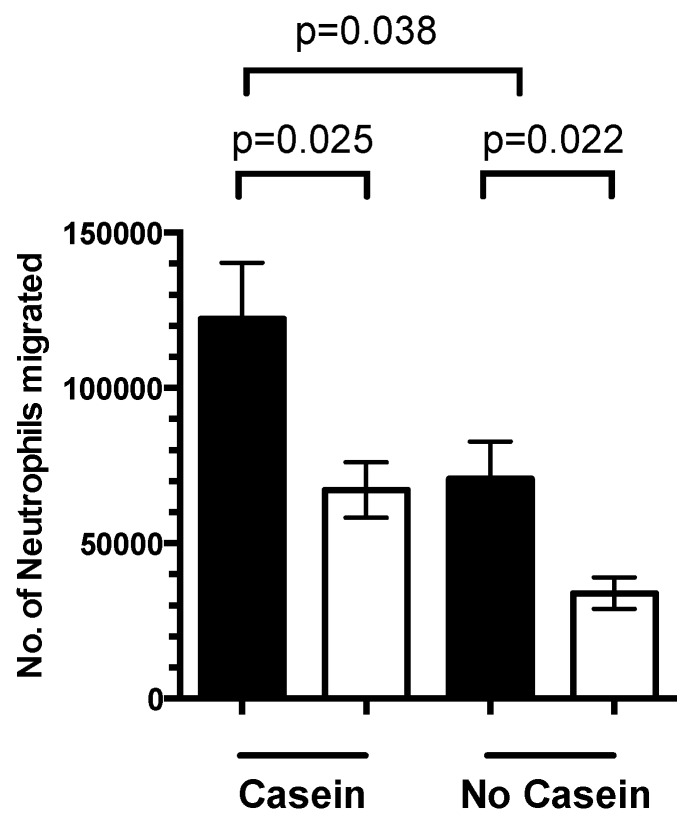
Effects of 1,25(OH)_2_D_3_ pre-treatment on neutrophil migration. A total of 4 × 10^6^ neutrophils were pre-treated with 100 nmol/L of 1,25(OH)_2_D_3_ for 1 h followed by 1 h stimulation with 5.2 mg/mL casein. Bars represent mean ± SEM (*n* = 7). Significance was calculated using a Student’s *t*-test (black bar = untreated; white bar = 1,25(OH)_2_D_3_).

We wanted to extend the above findings to examine the effect of 1,25(OH)_2_D_3_ on neutrophil migration. Pre-treatment of neutrophils with 1,25(OH)_2_D_3_ showed a 57% reduction in neutrophil migration in the absence of casein compared with untreated neutrophils (*p* = 0.022) (Figure 6). In casein-stimulated cells, pre-treatment with 1,25(OH)_2_D_3_ reduced neutrophil migration by 46% (*p* = 0.038) compared to untreated neutrophils.

## 4. Discussion

Vitamin D has a number of biological effects, including the modulation of critical immune responses involved in host protection. To date, very few studies have investigated this aspect, particularly in the context of bacterial infection. We investigated the effects of 1,25(OH)_2_D_3_ on ex vivo PBMCs stimulated with Gram-positive (pneumococci) or Gram-negative (LPS) bacterial ligands. In this study, we found that pre-treatment with 1,25(OH)_2_D_3_ reduced the levels of TNF-α, IFN-γ, IL-1β, and IL-8 in PBMC supernatants in response to heat-killed pneumococcal serotype 19F (HK19F) and LPS stimulation, while only TNF-α and IL-1β were reduced in monocytes.

These results support and extend the broader findings of 1,25(OH)_2_D_3_ inhibition of Th1 cytokine production during inflammation [20,21,22,23]. Both 1,25(OH)_2_D_3_ and 25(OH)D_3_ modulated cytokine responses of both PBMCs and purified monocytes, attributed to CYP27B1 expression on monocytes, macrophages and dendritic cells (DCs) that convert 25(OH)D_3_ into its functionally active form [24]. In monocytes, binding of 1,25(OH)_2_D_3_-activated VDR/retinoic X receptor (RXR) heterodimer to vitamin D response elements leads to modulation of key early response immune genes such as CD14 and IL-10, supporting its antimicrobial effects [25,26]. We used HK19F to mimic pneumococcal infection as serotype 19F is one of the most common serotypes that colonise children in developing countries, and is also a vaccine serotype [27]. Pneumococci HK19F represents the intact organism which signals through TLR2 and TLR4, respectively [28,29,30]. Other studies that used peptidoglycan from a non-encapsulated *S. pneumoniae* strain may not reflect true in vivo responses [19].

Lipopolysaccharide was also used to characterise pathogen specificity of vitamin D since 25(OH)D_3_ was previously shown to reduce cytokine levels in response to LPS, but not Pam3Cys (TLR2 ligand) [21]. Interestingly, IL-10, a regulatory cytokine, was increased by 1,25(OH)_2_D_3_ following HK19F but not LPS stimulation, suggesting a differential impact between these bacterial ligands. This effect for HK19F is noteworthy since excessive inflammation can lead to dissemination of pneumococci to sterile sites in the body as well as facilitate increased transmission [31,32]. Indeed, sustained high levels of TNF-α, IL-6 and IFN-γ are associated with increased disease severity during pneumococcal pneumonia [33]. The ability of 1,25(OH)_2_D_3_ to control inflammation through IL-10 is intriguing, and is consistent with its reported anti-inflammatory effects [34,35,36,37,38] and ability to induce functional regulatory T cells (Treg) [39,40]. Our data suggest this may be due to an increased CD14 expression although our small sample size limit the significance of this finding. Other reports have demonstrated that 1,25(OH)_2_D_3_ can skew DC-mediated T cell responses from an inflammatory Th1/Th17 phenotype to an anti-inflammatory Treg phenotype [19], possibly via epigenetic reprogramming [41]. This has relevance for enteric bacterial infections given the high proportion of Treg found in mucosal tissues [42] and that 25(OH)D_3_ deficiency is associated with low IL-10 levels [43]. Further studies on the role of vitamin D on Treg frequency and function are needed given their anti-inflammatory role.

The anti-inflammatory effects of 1,25(OH)_2_D_3_ were supported by our ex vivo data for HK19F where vitamin D-insufficient individuals had significantly higher IFN-γ than those who were sufficient, consistent with earlier data [44,45,46]. Down-regulation of pattern recognition receptor expression on innate immune cells by 1,25(OH)_2_D_3_ would be advantageous in the control of an excessive inflammatory environment [47]. However, during the initial stages of infection, 1,25(OH)_2_D_3_ may protect against bacterial infections by augmenting CD14 expression, as shown by our data from LPS-stimulated PBMCs. It has been shown that 1,25(OH)_2_D_3_ acts in a paracrine manner with human epithelial cells by up-regulating IL-1β secretion in *Mycobacterium tuberculosis*-infected macrophages [48]. Importantly, while vitamin D can dampen inflammatory responses, it does not seem to negatively impact protective immunity, since individuals with high plasma vitamin D levels (i.e., 25(OH)D) exhibited normal or enhanced responses to common bacterial and viral vaccines [49,50,51]. The vitamin D-VDR signal is also critical for T cell activation [52], providing a possible explanation for the increased ex vivo CD4 expression observed in our study.

We also found that 1,25(OH)_2_D_3_ reduces the migration capacity of neutrophils to casein, a well-characterised chemoattractant, demonstrating that 1,25(OH)_2_D_3_ can exert its effects on multiple cell targets critical for controlling inflammation [53,54]. It is known that 1,25(OH)_2_D_3_ enhances antimicrobial activity via interaction with VDR to up-regulate the synthesis of antimicrobial peptides such as cathelicidin and β-defensin [55,56]. Cathelicidin is produced by neutrophils, monocytes, and macrophages and has broad antimicrobial activity against bacteria, as well as viruses, such as respiratory syncytial virus (RSV) [57,58]. Moreover, under subclinical inflammatory conditions, vitamin D may be less able to control inflammatory responses in otherwise healthy individuals [59]. Further elucidation of these complex mechanisms are needed to understand how to harness these beneficial effects of vitamin D in humans.

Evidence for these effects of vitamin D from larger clinical settings has not been conclusive. Epidemiologic studies demonstrate strong associations between seasonal variations in 25(OH)D levels and influenza infection [10], as well as response to vaccines as a result of variable VDR expression profiles [60,61,62]. Elevated C-reactive protein as a marker of inflammation was inversely associated with 25(OH)D deficiency in newborns born in winter–spring, supporting this seasonal effect [63]. Moreover, dysbiosis of the microbiome and susceptibility to infection can be shaped by 25(OH)D status, further illustrating the biological importance of this molecule in maintaining health [64,65]. However, translating these findings to clinical trials of 1,25(OH)_2_D_3_ supplementation in infants and adults have not been promising largely due to the inconsistency among trial designs. Therefore, standardisation between clinical trials is critical to elucidate the role of 1,25(OH)_2_D_3_ in protection against bacterial infection.

Several limitations of our study need to be addressed. While our cohort of healthy adults had a spectrum of plasma 25(OH)D levels, we were still able to observe an effect upon 1,25(OH)_2_D_3_ treatment. Studies in known vitamin D (25(OH)D) deficient populations may reveal more potent effects in this context. Our sample size was relatively small and so further validation in larger clinically-relevant settings are needed. The examination of vitamin D to enhance protection during early life should be a priority, particularly where exposure to sun may be limited and the pathogen burden may be high. Our results support the continued investigation of vitamin D in randomized controlled trials, with potential effects having implications for vaccination, as well as development of novel therapies for infectious disease.

## 5. Conclusions

This study supports the anti-inflammatory effects of 1,25(OH)_2_D_3_ during bacterial infection. These results have important implications for the role of vitamin D in protection against infectious disease, particularly in high-risk settings involving low sunlight and increased exposure to pathogenic microorganisms. Further investigations, particularly in rigorous clinical trials, are required to understand to elucidate the beneficial effects of vitamin D in this context.
